# Laryngospasm during pulsed field ablation: A sign of warning for a device-specific complication?

**DOI:** 10.1016/j.hrcr.2025.09.007

**Published:** 2025-11-15

**Authors:** Łukasz Zarębski, John Mandrola, Piotr Futyma

**Affiliations:** 1 St. Joseph’s Heart Rhythm Center, Rzeszów, Poland; 2 University of Rzeszów, Rzeszów, Poland; 3 Baptist Health, Louisville, Kentucky

Rapidly increasing numbers of pulmonary vein isolation procedures for the treatment of atrial fibrillation have led to more frequent use of deep sedation and general anesthesia for pulsed field ablations (PFAs). Although most discussions on PFA safety have focused on its cardiac and extracardiac tissue selectivity, periprocedural airway management has received less attention. Nevertheless, constant ventilation remains critical during all pulmonary vein isolation procedures, given that even transient airway obstruction can have immediate consequences.

In this issue of *Heart Rhythm Case Reports*, Suga et al^1^ describe 3 cases of acute airway obstruction occurring during PFA performed under general anesthesia with a laryngeal mask airway (LMA). The clinical features were consistent with laryngospasm: airway obstruction developed abruptly after PFA delivery despite stable conditions beforehand, and attempts at repositioning the LMA were ineffective, whereas administration of neuromuscular blocking agents resulted in immediate resolution. These episodes occurred during ablation not only near the left superior pulmonary vein, where direct stimulation of the recurrent laryngeal nerve could be suspected, but also at other pulmonary vein sites, suggesting a more generalized mechanism.

The pathophysiological basis remains uncertain. PFA is known to induce transient skeletal muscle contractions, coughing, and diaphragmatic movement,^2^ which may destabilize airway positioning when an LMA is used. These movements can lead to LMA displacement and secondary upper-airway irritation, thereby precipitating laryngospasm. However, pulsed electric fields may directly stimulate recurrent laryngeal or vagal branches in proximity to the atrium and pulmonary veins or indirectly activate vagal afferents around the heart, bronchi, or esophagus. Both pathways could trigger reflex glottic closure via central neural circuits, analogous to phrenic nerve capture or vagal reflexes sometimes observed during PFA delivery.^3^ The reproducibility of these events across different ablation sites supports the hypothesis that laryngospasm may reflect a generalized reflex autonomic response to extracardiac neural stimulation.

From a clinical perspective, these reports raise important considerations for anesthetic management during PFA. LMAs are widely used in electrophysiology procedures because they are less invasive than endotracheal intubation and generally sufficient for thermal ablation.^4^ However, the specific physiological effects of PFA may increase the risk of reflex airway obstruction, particularly in the absence of ongoing neuromuscular blockade. In the present cases, laryngospasm occurred when neuromuscular blocking agents were absent or their effect had diminished, and resolution was achieved after administration of neuromuscular blocking agents. This observation suggests that if LMAs are used, maintenance of preventive neuromuscular blockade may be useful. Alternatively, in patients with an increased risk of airway complications—such as obesity, gastroesophageal reflux, or reactive airway disease—primary endotracheal intubation may provide greater safety. Continuous capnography remains essential, given that sudden loss of end-tidal carbon dioxide may be the earliest indication of obstruction and anesthesia teams should be ready for neuromuscular blockade and intubation if ventilation with LMA cannot be restored.

To date, laryngospasm has only been reported during procedures performed with the PulseSelect system (Medtronic).^5^ This system is adapted from the older pulmonary vein ablation catheter technology introduced nearly 2 decades ago, which delivers energy simultaneously through multiple splines, producing a relatively large atrial footprint. In the past, such catheter designs have been associated with reports of moderate pulmonary vein stenosis and frequent coughing during energy delivery, reflecting their less selective action on surrounding tissues.^6^^,^^7^ The broader anatomic exposure increases the likelihood of stimulating adjacent neural structures, including laryngeal reflex pathways. This risk is most likely specific to the PulseSelect design and should not be generalized for other PFA platforms, although systematic data are still required to determine whether catheter architecture influences susceptibility. A possible exception might be nonstandard scenarios such as the “olive strategy,” where the Farawave catheter (Farapulse Inc) is positioned more deeply within the pulmonary veins, potentially altering the stimulation profile.^8^^,^^9^ It is also reasonable to hypothesize that focal-bipolar PFA catheters, which deliver energy through smaller electrode surfaces, create lesions with more restricted spatial distribution, and are less dependent on pulmonary vein diameter, might reduce the chance of triggering such airway reflexes.^10^ However, at present, the frequency of these events remains unknown, and recognition relies largely on anecdotal reporting. Ongoing prospective studies may clarify whether such complications could also occur with newer focal-bipolar PFA systems (NCT07171463).

The cases presented by Suga et al^1^ extend our understanding of the safety profile of PFA by identifying laryngospasm as a new potential complication. Although rare, airway obstruction can have immediate and severe consequences. Future studies should estimate the frequency of this complication, clarify its exact mechanism, and establish optimal airway strategies for PFA.

## Disclosures

P.F. reports ventricular tachycardia ablation support for Abbott, consultations for Boston Scientific, and patent applications related to bipolar and high-voltage ablation. He also holds equity in CorSystem. Ł.Z. and J.M. declare no conflict.
